# VIEWPOINT FROM THE DIVISION OF AGING BIOLOGY

**DOI:** 10.1093/geroni/igz038.1639

**Published:** 2019-11-08

**Authors:** Felipe Sierra

**Affiliations:** National Institute on Aging, National Institutes of Health, Bethesda, Maryland, United States

## Abstract

Dr. Sierra will review accomplishments of the Division of Aging Biology and make projections of future growth in the field.

